# Stomatognathic Dysfunction and Neuropsychological Imbalance: Associations with Salivary Cortisol, EMG Activity, and Emotional Distress

**DOI:** 10.3390/dj13060230

**Published:** 2025-05-22

**Authors:** Ioana Scrobota, Liliana Sachelarie, Timea Claudia Ghitea, Georgiana Ioana Potra Cicalau, Roxana Alexandra Cristea, Pelea Diana, Petra Saitos, Alexandra Vlad, Loredana Liliana Hurjui

**Affiliations:** 1 Department of Dental Medicine, Faculty of Medicine and Pharmacy, University of Oradea, 10 1st Decembrie Street, 410073 Oradea, Romania; ioana_scrobota@uoradea.ro (I.S.); cristea.roxana.alexandra@didactic.uoradea.ro (R.A.C.); petrasaitos@uoradea.ro (P.S.); 2 Department of Preclinical Discipline, Faculty of Medicine, Apollonia University, 700511 Iasi, Romania; 3 Pharmacy Department, Faculty of Medicine and Pharmacy, University of Oradea, 1 University Street, 410087 Oradea, Romania; timea.ghitea@csud.uoradea.ro; 4 Preclinics Department, Faculty of Medicine and Pharmacy, University of Oradea, 10 1st Decembrie Street, 410073 Oradea, Romania; diana_pelea@uoradea.ro; 5 Doctoral School of Biomedical Sciences, Faculty of Medicine and Pharmacy, University of Oradea, 410087 Oradea, Romania; alvlad@uoradea.ro; 6 Morpho-Functional Sciences II Department, Faculty of Medicine, Discipline of Physiology, “Grigore T. Popa” University of Medicine and Pharmacy Iasi, 16 Universitatii Str., 700115 Iasi, Romania; loredana.hurjui@umfiasi.ro

**Keywords:** orofacial disorders, salivary cortisol, psychological distress

## Abstract

Stomatognathic dysfunctions, particularly bruxism, orofacial pain, and temporomandibular joint (TMJ) disorders, are frequently associated with anxiety and depression. However, the reverse hypothesis that these dysfunctions may act as peripheral stressors contributing to neuropsychological imbalance by activating the hypothalamic–pituitary–adrenal (HPA) axis remains underexplored. **Objective**: To assess the relationship between the severity of stomatognathic symptoms and both physiological and psychological stress markers to determine whether such dysfunctions may influence or exacerbate anxiety and depressive disorders. **Methods**: A cross-sectional observational study was conducted on 120 adult patients. Clinical assessment was included evaluation of TMJ function, bruxism, and orofacial pain. Psychological screening was performed using validated questionnaires (GAD-7 for anxiety and PHQ-9 for depression). Electromyographic (EMG) analysis of the masseter and temporalis muscles was conducted, along with the quantification of salivary cortisol using an enzyme-linked immunosorbent assay (ELISA). Multiple regression models were applied to identify statistically significant correlations (*p* < 0.05). **Results**: A positive correlation was found between the severity of stomatognathic dysfunction and scores on anxiety and depression measures, along with elevated salivary cortisol levels and increased masticatory muscle activity. **Conclusions**: These findings suggest a bidirectional relationship between stomatognathic dysfunctions and neuropsychological status, underscoring the potential value of integrated therapeutic approaches that combine dental care with psychological support.

## 1. Introduction

The stomatognathic system is crucial in essential functions, including mastication, speech, and facial expression. Beyond its mechanical and anatomical significance, this complex system has increasingly been linked to broader physiological and psychological processes. In recent years, researchers have begun to explore the bidirectional relationship between stomatognathic dysfunctions such as bruxism, temporomandibular joint (TMJ) disorders, chronic orofacial pain, and mental health conditions like anxiety and depression [1].

While the influence of stress and emotional distress on oral health is well documented, considerably less emphasis has been placed on the emerging hypothesis that stomatognathic dysfunctions such as bruxism, temporomandibular joint disorders, and chronic orofacial pain may themselves serve as contributing factors in the onset, maintenance, or exacerbation of neuropsychological imbalances, including anxiety and depression, through physiological stress pathways such as hyperactivity of the hypothalamic–pituitary–adrenal (HPA) axis [2].

Bruxism, defined as repetitive jaw muscle activity characterized by clenching or grinding of the teeth, has been widely associated with psychological stress and anxiety [1,2,3,4]. It is generally classified into two main types: awake bruxism, which is often semi-voluntary and associated with emotional states such as tension or frustration, and sleep bruxism, which is considered a sleep-related movement disorder [5,6,7,8,9]. Numerous studies have demonstrated a strong link between bruxism and elevated levels of anxiety, with psychological distress believed to trigger or exacerbate motor hyperactivity in the masticatory system [3,10].

According to Rollman et al. [7], individuals with high levels of negative affectivity and emotional dysregulation are more prone to bruxism, particularly under stressful conditions [11,12]. Bruxism has also been proposed as a maladaptive coping mechanism, acting as an unconscious outlet for unresolved emotional stress [13,14,15,16,17]. From a physiological perspective, emotional distress can activate the autonomic nervous system and the HPA axis, resulting in muscle hyperactivity, sleep disturbances, and increased cortisol secretion—all of which may contribute to bruxism episodes during both wakefulness and sleep [18,19].

Moreover, the prevalence of bruxism has been shown to increase in populations exposed to academic, occupational, or pandemic-related stress, suggesting that bruxism is not merely a mechanical or occlusal phenomenon but a complex biopsychosocial condition [15,20]. Recent studies also suggest that persistent bruxism may lead to secondary complications such as myofascial pain, temporomandibular joint damage, and impaired oral health-related quality of life [21].

These findings underscore the need to assess bruxism not only from a dental or functional perspective but also in conjunction with psychological screening, particularly in patients presenting with stress, anxiety, or depression symptoms [2]. However, studies also suggest that the prolonged activity of masticatory muscles and associated discomfort may act as peripheral stressors, contributing to systemic stress responses through the hypothalamic–pituitary–adrenal (HPA) axis [4].

Elevated salivary cortisol levels, a well-established biomarker for stress, have been observed in individuals with severe bruxism and temporomandibular joint (TMJ) disorders [4]. Cortisol, the end product of the hypothalamic–pituitary–adrenal (HPA) axis, plays a crucial role in the physiological stress response. When stress becomes chronic or poorly regulated, persistently high cortisol levels can affect various systems, including neuromuscular and immune pathways, thereby exacerbating or perpetuating musculoskeletal dysfunction [17].

Several studies have demonstrated that patients with myofascial pain and TMD present with higher morning salivary cortisol levels than control subjects, particularly when comorbid with anxiety or depression [18]. This suggests a bidirectional feedback loop in which chronic musculoskeletal tension, particularly in the masticatory muscles, triggers stress-related hormonal responses, which in turn intensify neuromuscular hyperactivity and pain perception.

Furthermore, cortisol itself may have a catabolic effect on muscle tissue, potentially contributing to muscle fatigue and an increased risk of dysfunction in individuals with sustained jaw activity, such as those with bruxism. Evidence from Mokhtari et al. [18] and Cao et al. [20] further supports this model, showing that psychological distress, salivary cortisol elevation, and functional muscle disorders tend to coexist in both clinical and at risk populations.

These findings highlight the importance of integrating salivary cortisol testing into the diagnostic process for patients with complex or recurrent stomatognathic complaints. Cortisol monitoring may offer insights into underlying stress physiology, enabling more targeted interventions addressing both psychological and somatic aspects of TMD and bruxism.

Temporomandibular disorders (TMDs) represent another significant component of stomatognathic dysfunction, often manifesting as joint clicking, restricted mouth opening, or facial pain. TMD is estimated to affect approximately 5–12% of the population and shows a notable prevalence among individuals with anxiety and depressive symptoms [6]. The chronic pain associated with TMD not only affects quality of life but also appears to influence emotional regulation and coping mechanisms, suggesting a deeper neurophysiological interaction [7].

Recent advances in diagnostic technology have enabled the assessment of masticatory muscle activity through electromyography (EMG), offering objective insights into the neuromuscular component of stomatognathic dysfunctions. EMG is a non-invasive, reliable tool that records electrical potentials generated by muscle fibers, allowing for the evaluation of both resting tone and dynamic contraction patterns in muscles such as the masseter and temporalis. It has proven particularly useful in identifying muscle hyperactivity, fatigue, and asymmetry—hallmarks of conditions like bruxism and temporomandibular disorders (TMDs) [8].

Studies have shown that patients with TMD often exhibit significantly elevated resting EMG activity compared to healthy controls, suggesting a state of chronic muscle tension or central sensitization [18]. Furthermore, EMG parameters have been correlated with subjective pain scores and emotional stress indicators, such as anxiety and depression levels [20]. This suggests that muscle overactivation may not merely be a mechanical consequence of malocclusion or parafunction but also a somatic expression of psychological distress.

The integration of EMG analysis in both research and clinical settings enables the quantification of neuromuscular dysfunction, allowing for objective monitoring over time and enhancing diagnostic accuracy when combined with clinical examinations and psychological assessments. In particular, increased EMG amplitudes in patients with high GAD-7 or PHQ-9 scores further support the hypothesis of a psychophysiological interaction affecting stomatognathic function [21,22,23].

Given its diagnostic value, EMG is increasingly recommended in the interdisciplinary evaluation of patients with suspected bruxism, myofascial pain, or stress-related oral motor disorders, offering a bridge between dentistry, neurology, and behavioral medicine [19].

The main objective of the study is to investigate the relationship between the severity of stomatognathic symptoms and physiological (salivary cortisol level and EMG activity) and psychological (anxiety and depression) markers, to determine whether these dysfunctions may contribute to the emergence or exacerbation of neuropsychological imbalances.

The present study adds value by simultaneously integrating objective measurements—electromyographic activity (EMG) and salivary cortisol levels—in the analysis of the relationship between stomatognathic function and psychological status.

The growing interest in psychoneuroimmunology and integrative care underscores the value of exploring oral–mental health connections for earlier diagnosis and multidisciplinary management.

This study proposes an integrated analytical model that correlates stomatognathic dysfunctions not only with self-reported anxiety and depression scores (GAD-7 and PHQ-9), but also with objective biological markers (salivary cortisol) and neuromuscular activity (EMG), aiming to determine whether these dysfunctions are merely consequences of emotional distress or, in certain contexts, may act as triggering factors influencing mental health through physiological stress pathways.

## 2. Materials and Methods

### 2.1. Study Design and Setting

This cross-sectional observational study was conducted over a 6-month period at Apollonia University in Iasi, Romania. The local Ethics Committee of Apollonia University from Iasi, Romania approved the study protocol (No. 68/16.10.2024) and adhered to the ethical standards outlined in the Declaration of Helsinki. All participants signed informed consent before enrollment.

### 2.2. Study Population

A total of 120 adult participants, aged between 18 and 60 years, were consecutively recruited over a six-month period from outpatient dental and psychological clinics affiliated with the study center. The recruitment process aimed to ensure a representative sample of individuals presenting with various degrees of stomatognathic dysfunction, as well as associated psychological symptoms.

The inclusion criteria required participants to present at least one clinical sign of stomatognathic dysfunction, as determined by clinical evaluation. Eligible signs included bruxism—either awake or sleep bruxism, evidenced by tooth wear facets, masseter muscle hypertrophy, or self-reported clenching and grinding; temporomandibular joint disorder (TMD), diagnosed according to the Diagnostic Criteria for Temporomandibular Disorders (DC/TMD), manifested by joint sounds, pain on palpation, or limited mandibular movement; and chronic orofacial pain, defined as pain persisting for more than three months, reported either spontaneously or during clinical examination. Additionally, participants were required to be capable of providing informed consent and to be free from systemic neurological or endocrine disorders that could potentially confound the study outcomes.

The exclusion criteria included current use of anxiolytic or antidepressant medication, ongoing orthodontic treatment, a history of craniofacial trauma, pregnancy, and systemic conditions known to influence neuromuscular or hormonal parameters. These exclusions were applied to minimize confounding variables and to enhance the reliability of correlations between stomatognathic, physiological, and psychological factors.

### 2.3. Psychological Assessment

Participants were assessed using two validated questionnaires as assessment instruments. The GAD-7 (Generalized Anxiety Disorder Scale—7 items) is a screening instrument used to identify symptoms of generalized anxiety, in which scores ≥ 10 indicate a moderate to severe level of anxiety.

We used the validated Romanian versions of the GAD-7 and PHQ-9 questionnaires, according to the study conducted by Rogoveanu et al. [24], which demonstrated high internal consistency and clinical applicability in the Romanian population (Cronbach coefficient ≈ 0.89).

The PHQ-9 (Psychological Health Questionnaire—9 items) was also applied, an instrument designed to identify depressive symptoms, in which scores ≥ 10 are considered suggestive of the presence of significant clinical depression.

The questionnaires were completed in a quiet environment, under the supervision of a clinical psychologist, to minimize external influences and to ensure that participants correctly understood each item.

### 2.4. Clinical Evaluations

All participants received a comprehensive clinical evaluation targeting the functional, muscular, and psychological dimensions of stomatognathic dysfunction. The diagnostic process began with an intraoral and extraoral examination to assess signs of bruxism, including tooth wear facets, hypertrophy of the masseter muscles, and the patient’s reported clenching or grinding habits. Bruxism was assessed clinically based on tooth wear facets, hypertrophy of the masseter muscles, and self-reported clenching or grinding habits. These criteria have been described in the literature as reliable clinical indicators of bruxism and are widely accepted for diagnostic purposes when instrumental methods are not available [22].

Tooth wear was assessed clinically by visual examination of wear facets on occlusal and incisal surfaces during intraoral examination. No standardized wear index was applied; instead, the extent and severity of wear were categorized based on clinical judgment into mild, moderate, or severe.

Hypertrophy of the masseter muscles was evaluated through bilateral palpation and visual inspection during maximal voluntary clenching (MVC), with increased muscle prominence and firmness relative to normative expectations considered indicative of hypertrophy.

Orofacial pain intensity was measured using a visual analog scale (VAS) ranging from 0 (no pain) to 10 (severe pain), with both spontaneous and palpation-induced discomfort being recorded. Palpation was systematically performed on bilateral masticatory muscles, including the masseter, temporalis, and medial pterygoid muscles, following standardized clinical examination protocols.

All clinical assessments, including the evaluation of bruxism, temporomandibular joint function, and orofacial pain, were conducted by a specialist in orofacial pain with clinical expertise in temporomandibular disorders, ensuring diagnostic accuracy and methodological consistency throughout the study.

### 2.5. Electromyographic (EMG) Analysis

Electromyographic (EMG) activity of the masseter and anterior temporalis muscles was recorded using surface electrodes and a specific EMG device (TrueTrace EMG, Deymed Diagnostic, Hronov, Czech Republic, distributed by Hellimed, Bucharest, Romania). Surface electrodes were positioned bilaterally following standard anatomical landmarks, and EMG signals were recorded under two conditions: at rest and during maximum voluntary clenching (MVC) on cotton rolls. The muscle tone, symmetry index, and hyperactivity patterns were analyzed to quantify neuromuscular imbalance.

### 2.6. Salivary Cortisol Analysis

Unstimulated whole saliva samples were collected between 8:00 and 9:00 a.m. using the passive drool method, in accordance with established protocols for assessing salivary biomarkers of physiological stress [4,18]. To ensure standardization and avoid contamination or variability in biomarker concentrations, participants were instructed to abstain from eating, drinking (except water), smoking, or performing oral hygiene procedures for at least 60 min prior to sample collection. Each participant provided approximately 2 milliliters of saliva, which was immediately stored at −20 °C to preserve the biochemical integrity of the samples until further analysis.

Salivary cortisol concentrations were determined using a high-sensitivity enzyme-linked immunosorbent assay (ELISA) kit (Salimetrics LLC, State College, PA, USA), following the manufacturer’s instructions. All samples were analyzed in duplicate, and both intra-assay and inter-assay coefficients of variation were kept below 10%, ensuring the acceptable reliability and reproducibility of the results.

Cortisol levels were expressed in nanomoles per liter (nmol/L). Elevated values were interpreted as reflective of an increased physiological stress response, potentially associated with heightened hypothalamic–pituitary–adrenal (HPA) axis activity.

### 2.7. Statistical Analysis

Descriptive statistics were calculated for the demographic, clinical, and psychological variables. The normality of data distribution was assessed using the Shapiro–Wilk test. Depending on the distribution, Pearson’s or Spearman’s correlation coefficients were used to explore associations between salivary cortisol levels, EMG findings, and GAD-7 and PHQ-9 scores. Differences in clinical variables between groups with high versus low psychological distress were assessed using *t*-tests or Mann–Whitney U tests, as appropriate. Multiple linear regression models were employed to identify independent predictors of anxiety and depression, including stomatognathic dysfunction severity, muscle hyperactivity, and cortisol concentration as covariates. A *p*-value < 0.05 was considered statistically significant. All data were analyzed using SPSS software version 25.

## 3. Results

### 3.1. Demographic Characteristics

A total of 120 participants were included in the study, with a mean age of 35.6 ± 10.2 years (Table 1). The gender distribution indicated a higher proportion of female participants (*n* = 74; 61.7%) compared to males (*n* = 46; 38.3%), consistent with the reported higher prevalence of temporomandibular disorders (TMD) and bruxism among women in the previous literature [1].

Bruxism was clinically identified in 67 individuals, representing 55.8% of the sample, while signs of TMD were found in 58 participants (48.3%). These findings confirm the frequent coexistence of bruxism and TMD in the general population.

The average pain intensity, measured using the Visual Analog Scale (VAS), was 4.8 ± 2.1, indicating a moderate level of orofacial discomfort. Psychological screening revealed a mean GAD-7 score of 9.2 ± 5.0 and a mean PHQ-9 score of 8.7 ± 4.6, suggesting that a substantial proportion of participants experienced at least mild to moderate levels of anxiety and depressive symptoms.

Physiological stress, as measured by salivary cortisol, showed a mean concentration of 15.3 ± 6.1 nmol/L. These values are within the upper normal range for morning cortisol but may reflect heightened hypothalamic–pituitary–adrenal (HPA) axis activity in participants with combined stomatognathic and psychological symptoms.

The mean resting EMG activity recorded in the masseter and temporalis muscles was 7.4 ± 2.8 μV, which is considered elevated according to previous studies that reported normal resting EMG values in healthy individuals generally ranging between 2 and 5 μV [23].

Taken together, these findings underscore the interplay between stomatognathic dysfunctions, psychological distress, and heightened physiological stress markers within the study population, emphasizing the necessity for integrated diagnostic and therapeutic approaches.

### 3.2. Correlation and Regression Analysis

Statistical analysis revealed significant correlations between physiological, psychological, and neuromuscular variables, Table 2.

A moderate positive correlation was observed between GAD-7 anxiety scores and salivary cortisol levels (r = 0.42, *p* < 0.001), indicating that higher anxiety symptoms were associated with an increased physiological stress response. Similarly, PHQ-9 depression scores correlated positively with cortisol concentrations (r = 0.38, *p* = 0.002), supporting the hypothesis of hypothalamic–pituitary–adrenal (HPA) axis activation in depressive states, Table 3.

Electromyographic (EMG) activity in the masseter and temporalis muscles was also positively associated with both GAD-7 (r = 0.36, *p* = 0.004) and PHQ-9 (r = 0.31, *p* = 0.011) scores. Participants with higher anxiety and depression symptoms exhibited elevated baseline muscle activity, suggesting a potential neuromuscular manifestation of psychological distress.

This table presents the independent predictors of psychological scores (GAD-7 and PHQ-9) based on multiple linear regression analysis. Each row displays the association between a single clinical predictor and the psychological outcome, including the standardized beta coefficient (β) and the *p*-value for statistical significance (*p* < 0.05 indicates a statistically significant predictor).

In the multiple linear regression analysis, salivary cortisol (β = 0.29, *p* = 0.006) and EMG activity (β = 0.24, *p* = 0.014) emerged as significant independent predictors of GAD-7 scores, after adjusting for age and gender (model R^2^ = 0.31, F = 7.14, *p* < 0.001). For PHQ-9 scores, the strongest predictors were pain intensity (VAS) (β = 0.33, *p* = 0.002) and salivary cortisol (β = 0.28, *p* = 0.009) (model R^2^ = 0.28, F = 6.21, *p* < 0.001).

These findings support the presence of a bidirectional relationship between stomatognathic dysfunctions and psychological stress. Notably, both muscular hyperactivity and increased salivary cortisol were associated with elevated levels of anxiety and depression, suggesting that the stomatognathic system may not only reflect but also contribute to neuropsychological imbalance through physiological stress pathways.

To further explore the clinical implications of these findings, participants were divided into subgroups based on GAD-7 scores (≥10 indicating moderate to severe anxiety). Independent *t*-tests revealed significantly higher EMG activity and salivary cortisol levels in the high anxiety group compared to those with minimal symptoms (*p* < 0.01). These results support the hypothesis that psychological distress is associated with increased neuromuscular and physiological stress responses within the stomatognathic system.

This table compares key physiological and clinical parameters between participants with low anxiety (GAD-7 < 10) and those with moderate to severe anxiety (GAD-7 ≥ 10). Values are presented as mean ± standard deviation. *p*-values are based on independent samples *t*-tests.

Table 4 highlights significant differences between participants with low (GAD-7 < 10; *n* = 75) and high anxiety levels (GAD-7 ≥ 10; *n* = 45). Individuals with higher anxiety showed elevated EMG activity (8.2 ± 2.4 µV vs. 6.5 ± 2.7 µV; *p* = 0.001), suggesting increased neuromuscular tension.

Salivary cortisol levels were also significantly higher (17.5 ± 5.8 vs. 13.2 ± 5.4 nmol/L; *p* < 0.001), indicating an enhanced physiological stress response. Additionally, pain intensity was more significant in the high anxiety group (VAS: 5.4 ± 2.2 vs. 4.1 ± 1.9; *p* = 0.009).

These results support the link between psychological distress and both physiological and functional alterations in the stomatognathic system.

## 4. Discussion

The findings of this study further support the bidirectional relationship between stomatognathic dysfunctions and psychological distress. Elevated anxiety and depression symptoms were associated with increased EMG activity of the masticatory muscles, higher pain intensity, and elevated salivary cortisol levels. These results align with the recent literature indicating that emotional distress contributes to neuromuscular tension and altered physiological function within the stomatognathic system [2].

The significant elevation in salivary cortisol among participants with higher GAD-7 scores is consistent with previous studies reporting heightened hypothalamic–pituitary–adrenal (HPA) axis activity in patients with temporomandibular disorders (TMDs) and anxiety [16,17]. Yoshida also emphasized the role of peripherally induced movement disorders following dental interventions, which may be worsened by pre-existing psychological stress [18].

The EMG hyperactivity in patients with high anxiety echoes findings from Mokhtari et al. [18], who demonstrated similar neuromuscular patterns in students under academic and emotional pressure [18,19]. Moreover, Yap et al. [19] highlighted the significant role of stress and negative affectivity in somatic symptoms and orofacial dysfunctions, reinforcing the observed EMG and pain relationships [21,23].

The link between pain perception and psychological state was evident in our results, as patients with higher anxiety levels also reported significantly greater pain intensity. This result aligns with the earlier conclusions of Rollman and Gillespie [7], who highlighted the influence of psychosocial factors including anxiety, depression, and somatization on pain sensitivity and their role in the persistence and worsening of orofacial pain over time [12]. In line with this, recent work by Cao et al. [20] demonstrated that both acute and chronic subtypes of temporomandibular disorders (TMDs) are not only mechanically driven but are significantly influenced by psychological distress and sleep disturbances.

Pain perception extends beyond sensory input, being shaped by cognitive-emotional factors such as fear, worry, and catastrophizing, which can lower thresholds and heighten somatic sensations through central sensitization [19]. As such, the increased VAS pain scores in patients with high anxiety in our study likely reflect a complex psychoneurological interaction rather than localized tissue damage alone.

Moreover, the psychological burden of TMD has been shown to affect patients’ quality of life. Yap et al. [19] highlighted that individuals with higher emotional distress and more severe TMD symptoms reported significantly lower oral health-related quality of life scores, particularly in areas related to daily functioning, social interactions, and emotional well-being. These effects were observed across diverse cultural settings, suggesting a universal pattern in how emotional dysregulation impacts the experience of pain and its broader consequences on the quality of life [23].

Taken together, these findings underscore the need to address both the physical and emotional aspects of TMD in clinical practice, emphasizing individualized, multidisciplinary treatment strategies that include psychological support where indicated.

Importantly, our regression analysis identified salivary cortisol and EMG activity as independent predictors of anxiety scores, suggesting a physiological pathway linking stomatognathic stress and neuropsychological imbalance. This aligns with Slavicek and Sato [13]’s concept of stress-related parafunctions and recent discussions on integrating psychological evaluation into TMD diagnostics. According to Slavicek and Sato [13], parafunctional behaviors such as bruxism, clenching, and jaw muscle hyperactivity are not merely mechanical habits but rather subconscious responses to psychological stress that become embedded in the neuromuscular system over time. These stress-induced parafunctions represent an adaptive coping strategy through which emotional tension is somatically expressed [13].

Building on this paradigm, recent authors have emphasized the need to routinely assess psychological status in patients presenting with orofacial pain, mainly when symptoms are chronic or unexplained by structural pathology. Tools such as the GAD-7 and PHQ-9 can provide valuable insights into a patient’s emotional state, guiding clinicians toward more comprehensive management strategies [24,25]. The integration of psychological screening is increasingly recognized as a cornerstone of contemporary TMD diagnostics, supporting a shift toward a biopsychosocial model that accounts for the interplay between mental health, neuromuscular function, and subjective symptom experience.

Incorporating psychological assessments not only enhances diagnostic precision but may also help prevent overtreatment with invasive or irreversible dental procedures in patients whose symptoms stem primarily from emotional dysregulation.

From a clinical perspective, our findings underscore the importance of a multidisciplinary approach in managing orofacial disorders, particularly in patients presenting with comorbid emotional symptoms. Early identification of psychological stress using tools such as the GAD-7 and salivary biomarkers may facilitate the development of more effective and individualized treatment strategies.

However, the present study has certain limitations. Its cross-sectional design does not permit causal inference, and the sample was drawn from a single geographic region, which may limit the generalizability of the findings. Additionally, several potential confounding variables—such as physical activity levels, sleep duration, caffeine intake, and other lifestyle factors—were not assessed and may have influenced both psychological and physiological parameters. Future longitudinal studies and controlled clinical trials are warranted to validate these associations and to determine whether targeted interventions for psychological distress can directly influence stomatognathic outcomes.

Clinicians treating patients with bruxism or TMD should consider psychological screening as part of routine diagnostic protocols. The use of brief, validated tools such as the GAD-7 and PHQ-9, combined with non-invasive physiological measurements (EMG and salivary cortisol), offers a promising integrative approach.

Further research is recommended to address the current study’s limitations and to strengthen the evidence regarding the bidirectional relationship between stomatognathic dysfunctions and psychological stress, particularly by exploring the effects of cognitive behavioral therapy (CBT), biofeedback, and mindfulness-based stress reduction (MBSR) on both psychological and stomatognathic parameters in randomized controlled trials.

## 5. Conclusions

This study reinforces the existence of a strong association between stomatognathic dysfunctions and psychological distress, particularly anxiety and depression. The results demonstrate that increased electromyographic (EMG) activity, elevated salivary cortisol levels, and greater pain intensity are significantly linked to higher GAD-7 and PHQ-9 scores. Both EMG activity and salivary cortisol were identified as independent predictors of anxiety, while pain intensity was more closely associated with depressive symptoms.

These findings support that the stomatognathic system is not only affected by psychological states but may also contribute to their development through neurophysiological pathways. The integration of psychological screening with physiological markers, such as salivary cortisol and EMG, may offer valuable insights for early diagnosis and tailored interventions. An interdisciplinary approach encompassing dental, psychological, and physiological perspectives is essential for the effective management of stress-related orofacial conditions.

## Figures and Tables

**Table 1:** Demographic and clinical characteristics of the study sample.

Variable	Total Sample (*n* = 120)
Age (years)	35.6 ± 10.2
Gender (female/male)	74/46
Presence of bruxism (%)	67 (55.8%)
Presence of TMD (%)	58 (48.3%)
Mean VAS pain score	4.8 ± 2.1
Mean GAD-7 score	9.2 ± 5.0
Mean PHQ-9 score	8.7 ± 4.6
Mean salivary cortisol (nmol/L)	15.3 ± 6.1
Mean EMG activity (µV)	7.4 ± 2.8

**Table 2:** Correlation coefficients between clinical and psychological variables.

Variables	Correlation Coefficient (r)	*p*-Value
GAD-7 vs. salivary cortisol	0.42	<0.001
PHQ-9 vs. salivary cortisol	0.38	0.002
GAD-7 vs. EMG activity	0.36	0.004
PHQ-9 vs. EMG activity	0.31	0.011

**Table 3:** Multiple linear regression analysis: independent predictors of psychological scores.

Psychological Outcome	Clinical Predictor	Beta Coefficient (β)	*p*-Value
GAD-7 Score	Salivary cortisol	0.29	0.006
GAD-7 Score	EMG activity	0.24	0.014
PHQ-9 Score	Pain intensity (VAS)	0.33	0.002
PHQ-9 Score	Salivary cortisol	0.28	0.009

**Table 4:** Comparative analysis between low and high anxiety groups (based on GAD-7 score).

Variable	Low Anxiety (GAD-7 < 10, *n* = 75) Mean ± SD	High Anxiety (GAD-7 ≥ 10, *n* = 45) Mean ± SD	*p*-Value
EMG activity (µV)	6.5 ± 2.7	8.2 ± 2.4	0.001
Salivary cortisol (nmol/L)	13.2 ± 5.4	17.5 ± 5.8	<0.001
VAS pain score	4.1 ± 1.9	5.4 ± 2.2	0.009

## Data Availability

The original contributions presented in this study are included in the article. Further inquiries can be directed to the corresponding authors.

## References

[1] Wieckiewicz M., Smardz J., Martynowicz H., Wojakowska A., Mazur G., Winocur E. (2020). Distribution of Temporomandibular Disorders among Sleep Bruxers and Non-Bruxers—A Polysomnographic Study. J. Oral Rehabil..

[2] Yoshida K. (2024). Movement disorders of the stomatognathic system: A blind spot between dentistry and medicine. Dent. Med. Probl..

[3] Manfredini D., Winocur E., Guarda-Nardini L., Paesani D., Lobbezoo F. (2013). Epidemiology of bruxism in adults: A systematic review of the literature. J. Orofac. Pain.

[4] dos Santos E.A., Peinado B.R.R., Frazão D.R., Né Y.G.S., Fagundes N.C.F., Magno M.B., Maia L.C., Lima R.R., de Souza-Rodrigues R.D. (2022). Association between Temporomandibular Disorders and Anxiety: A Systematic Review. Front. Psychiatry.

[5] Reissmann D.R., John M.T., Schierz O., Sierwald I., Sobottka A., Hirsch C. (2012). Stress-related adaptive versus maladaptive coping and temporomandibular disorder pain. J. Orofac. Pain.

[6] Slade G.D., Fillingim R.B., Sanders A.E., Bair E., Greenspan J.D., Ohrbach R. (2013). Summary of findings from the OPPERA prospective cohort study of incidence of first-onset temporomandibular disorder: Implications and future directions. J. Pain.

[7] Rollman G.B., Gillespie J.M. (2000). The role of psychosocial factors in temporomandibular disorders. Curr. Rev. Pain.

[8] Ferrando M., Andreu Y., Galdón M.J., Durá E., Poveda R., Boscá M.M. (2004). Psychological variables and temporomandibular disorders: Distress, coping, and personality. Oral Surg. Oral Med. Oral Pathol. Oral Radiol. Endod..

[9] Koecklin K.H.U., Aliaga-Del Castillo A., Li P. (2024). The neural substrates of bruxism: Current knowledge and clinical implications. Front. Neurol..

[10] Priyadharshini G., Ramalingam K., Ramani P. (2024). Unveiling the Unspoken: Exploring Oral Manifestations of Psychological Disorders. Cureus.

[11] Fernandes G., Gonçalves D.A., de Godoi Gonçalves D.A., Camparis C.M., Speciali J.G. (2013). Temporomandibular disorders, mood disorders, and muscle pain: A correlation study. J. Appl. Oral Sci..

[12] Wan J., Lin J., Zha T., Ciruela F., Jiang S., Wu Z., Fang X., Chen Q., Chen X. (2025). Temporomandibular disorders and mental health: Shared etiologies and treatment approaches. J. Headache Pain.

[13] Slavicek R., Sato S. (2014). Bruxism: The stress-related chewing motor disorder. Int. J. Prosthodont..

[14] AlSahman L., AlBagieh H., AlSahman R. (2023). Association of stress, anxiety and depression with temporomandibular disorders in young adults—A systematic review. Arch. Med. Sci..

[15] Yoshida K. (2023). Bruxism or Dystonia: That Is the Question. Perspectives.

[16] Yoshida K. (2022). Botulinum Toxin Therapy for Oromandibular Dystonia and Other Movement Disorders in the Stomatognathic System. Toxins.

[17] Yoshida K. (2023). Peripherally induced movement disorders in the stomatognathic system after oral surgical or dental procedures: A retrospective study of 229 cases. J. Oral Maxillofac. Surg. Med. Pathol..

[18] Mokhtari Z., Mohammadi H., Golkari A. (2020). The impact of stress, anxiety and depression on stomatognathic system of physiotherapy and dentistry first-year students. J. Clin. Exp. Dent..

[19] Yap A.U., Sultana R., Natu V.P. (2022). Stress and emotional distress: Their associations with somatic and temporomandibular disorder-related symptoms. Psychol. Health Med..

[20] Cao Y., Yap A.U., Lei J., Zhang M.J., Fu K.Y. (2021). Subtypes of acute and chronic temporomandibular disorders: Their relation to psychological and sleep impairments. Oral Dis..

[21] Yap A.U., Lee D.Z.R., Marpaung C. (2022). Negative affectivity and emotions in youths with temporomandibular disorders across cultures. J. Craniomandib. Sleep Pract..

[22] Lobbezoo F., Ahlberg J., Glaros A.G., Kato T., Koyano K., Lavigne G.J., Winocur E. (2013). Bruxism defined and graded: An international consensus. J. Oral Rehabil..

[23] Ferrario V.F., Sforza C., Serrao G., Dellavia C., Tartaglia G.M. (2004). Single tooth bite forces in healthy young adults. J. Oral Rehabil..

[24] Snijkers J.T.W., van den Oever W., Weerts Z.Z.R.M., Vork L., Mujagic Z., Leue C., Hesselink M.A.M., Kruimel J.W., Muris J.W.M., Bogie R.M.M. (2021). Examining the optimal cutoff values of HADS, PHQ-9 and GAD-7 as screening instruments for depression and anxiety in irritable bowel syndrome. Neurogastroenterol. Motil..

[25] Maślak-Bereś M., Loster J.E., Wieczorek A., Loster B.W. (2019). Evaluation of the psychoemotional status of young adults with symptoms of temporomandibular disorders. Brain Behav..

